# Supplementary material for: Towards chiral acoustoplasmonics
Source: Nanophotonics. 2023 Apr 28;12(11):1957–64. doi: 10.1515/nanoph-2022-0780 (PMC10193267; doi:10.1515/nanoph-2022-0780)
Supplement: Supplementary file 1 — Supplementary Material Details [file j_nanoph-2022-0780_suppl_001.pdf]

B. Castillo López de Larrinzar[1], C. Xiang[2], E. Cardozo de Oliveira[2],
N. D. Lanzillotti-Kimura[2,*], A. García-Martín[1,+]

Towards chiral acoustoplasmonics

Supplementary information

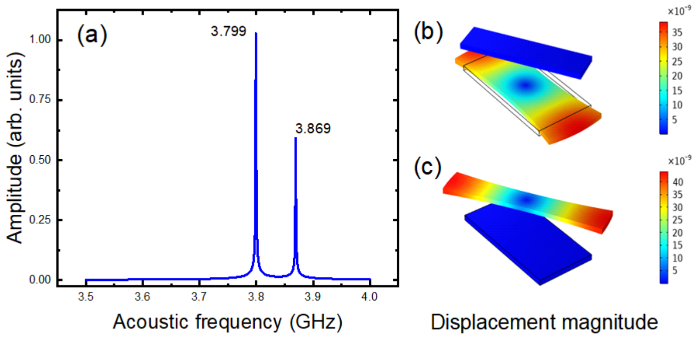

Figure S1: (a) Acoustic spectrum of the two twisted nanobars. The acoustic modes of the nanobars are obtained using the finite element method solver COMSOL Multiphysics. A thermal expansion due to a 5 K temperature rise is applied to both bars, which results in a localized strain in the bars. Notice that in this simulation we consider the case of two twisted bars without any solid environment. The frequency domain study calculates the displacements in all directions of the two bars between frequencies of 3.5 to 4 GHz, with a step of 0.001 GHz. Two distinct peaks in the result correspond to the eigenmodes of the two bars. Since the bars are not connected by any solid medium, each one of the modes is fully localized in a single bar. The slight difference in the frequency is due to the lateral confinement.

[1] Instituto de Micro y Nanotecnología IMN-CNM, CSIC, CEI UAM + CSIC, Isaac Newton 8, Tres Cantos, Madrid 28760, Spain
[2] Université Paris-Saclay, CNRS, Centre de Nanosciences et de Nanotechnologies, 10 Boulevard Thomas Gobert, 91120 Palaiseau, France
*email : daniel.kimura@c2n.upsaclay.fr
+email : a.garcia.martin@csic.es

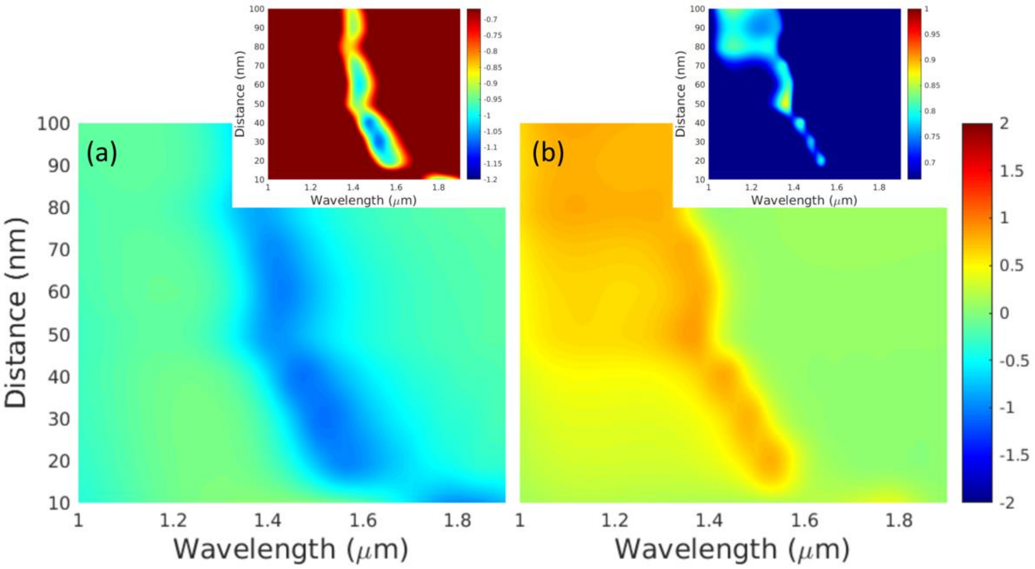

Figure S2: Figure of merit of the "asymmetry factor" (g), defined as ($\sigma^{R,L}_{Sc,Ab}$ stands for the Scattering or Absorption cross section for RCP or LCP):

$$g_{Sc,Ab} = 2\frac{\sigma^{R}_{Sc,Ab} - \sigma^{L}_{Sc,Ab}}{\sigma^{R}_{Sc,Ab} + \sigma^{L}_{Sc,Ab}}.$$

The value of g is bound between -2 and 2 for the extreme case where one of them is identically zero. Note that for a situation where one cross-section doubles the other $\sigma^{R} = 2\sigma^{L}$ then g=2/3 and if $2\sigma^{R} = \sigma^{L}$ then g=-2/3. In the spectral region of interest, for absorption (a) the prevalent polarization is LCP, whereas for scattering it is RCP (b), as already grasped from Figs. 2 and 3 in the main text. The larger the separation the shorter the wavelength where the maximum asymmetry occurs. In the insets we have saturated the false color maps to show the regions where the asymmetry is either smaller than -2/3 or larger than 2/3, evidencing ratios between the two polarizations larger than 100%. As depicted the sweet region where both confluence is for distances ca. D=60nm and wavelengths ca. 1360nm.
